# Clinical outcomes and prognostic factors of cyberknife stereotactic body radiation therapy for unresectable hepatocellular carcinoma

**DOI:** 10.1186/s12885-016-2512-x

**Published:** 2016-07-12

**Authors:** Jenny Que, Hsing-Tao Kuo, Li-Ching Lin, Kuei-Li Lin, Chia-Hui Lin, Yu-Wei Lin, Ching-Chieh Yang

**Affiliations:** Department of Radiation Oncology, Chi Mei Medical Center, No.901, Zhonghua Road, Yongkang district Tainan, 710 Taiwan; Department of Internal Medicine, Division of Hepatogastroenterology, Chi Mei Medical Center, Tainan, Taiwan

**Keywords:** Cyberknife, Stereotactic body radiation therapy, Hepatocellular carcinoma

## Abstract

**Background:**

Stereotactic body radiation therapy (SBRT) has been an emerging non-invasive treatment modality for patients with hepatocellular carcinoma (HCC) when curative treatments cannot be applied. In this study, we report our clinical experience with Cyberknife SBRT for unresectable HCC and evaluate the efficacy and clinical outcomes of this highly sophisticated treatment technology.

**Methods:**

Between 2008 and 2012, 115 patients with unresectable HCC treated with Cyberknife SBRT were retrospectively analyzed. Doses ranged from 26 Gy to 40 Gy were given in 3 to 5 fractions for 3 to 5 consecutive days. The cumulative probability of survival was calculated according to the Kaplan-Meier method and compared using log-rank test. Univariate and multivariate analysis were performed using Cox proportional hazard models.

**Results:**

The median follow-up was 15.5 months (range, 2-60 months). Based on Response Evaluation and Criteria in Solid Tumors (RECIST). We found that 48.7 % of patients achieved a complete response and 40 % achieved a partial response. Median survival was 15 months (4-25 months). Overall survival (OS) at 1- and 2-years was 63.5 %(54-71.5 %) and 41.3 % (31.6-50.6 %), respectively, while 1- and 2- years Progression-free Survival (PFS) rates were 42.8 %(33.0-52.2 %) and 38.8 % (29.0-48.4 %). Median progression was 6 months (3-16 months). In-field recurrence free survival at 1 and 2 years was 85.3 % (76.2-91.1 %) and 81.6 % (72.2-88.6 %), respectively, while the 1- and 2-years out-field recurrence free survival were 52.5 % (41.2-60.8 %) and 49.5 %(38.9-59.2 %), respectively. Multivariate analysis revealed that Child-Pugh score (A vs. B), Portal vein tumor thrombosis (positive vs. negative), Tumor size (≤4 cm vs >4-9 cm /≥10 cm), and tumor response after SBRT (CR vs. PR/stable) were independent predictors of OS. Acute toxicity was mostly transient and tolerable.

**Conclusions:**

Cyberknife SBRT appears to be an effective non-invasive treatment for local unresectable HCC with low risk of severe toxicity. These results suggested that Cyberknife SBRT can be a good alternative treatment for unresectable HCC unsuitable for standard treatment.

## Background

Hepatocellular carcinoma (HCC) is the sixth most common cancer and the third most common cause of cancer-related death worldwide [1]. Surgical resection, liver transplant, or radiofrequency ablation for the treatment of tumors ≤ 3 cm are the only curative treatment [2, 3]. Only a minority of patients are candidates for these treatments due to multifocal intrahepatic recurrence, extrahepatic extension, major vascular invasion, or impaired liver function caused by underlying cirrhosis. For patients not suitable for curative treatment, TACE was the most common alternative treatment. Although it does not completely eradicate HCC, it is an effective palliative regimen with improved survival compared with the best supportive care. However, for large (≥5 cm) or multiple tumors, HCC with portal vein thrombosis, and extrahepatic metastasis, TACE is less effective [4, 5]. For these patients, the use of sorafenib can increase 1-year survival to 45 %. The SHARP (Sorafenib hepatocellular Carcinoma Assessment Randomized Protocol) trial used sorafenib, a multikinase inhibitor, as an effective systemic treatment for advanced HCC, conferring an improvement in median survival of 2.8 months compared with placebo. However, invariable progression of the lesions was found among the patients treated with TACE or sorafenib [6, 7].

Historically, radiation therapy (RT) was not recommended for HCC patients because of the low tolerance of the liver to radiation and the difficulty in localizing tumors as a result of organ motion. However, with recent technological advancements such as stereotactic body radiation therapy (SBRT) and image-guided radiotherapy, tumoricidal doses can be delivered safely to the focal HCC while sparing the normal liver. Previously published data have yielded promising results, achieving high local control and acceptable rates of radiation-related toxicity [8, 9]. Although SBRT in the management of HCC has been increasingly recognized, there remain several questions to be answered. One of these involves the identification of prognostic factors to better understand and improve the outcome of SBRT for HCC.

Cyberknife robotic radiotherapy (Accuray Inc, Sunnyvale, CA, USA) with internal fiducial markers and synchrony respiratory tracking capabilities allows more accurate targeting by reducing the margin of error and normal tissue exposure during therapy and therefore increases the chances of treating larger tumors with limited normal liver volume available or tumors are in close proximity to critical organs. Cyberknife is a frameless whole-body image guided robotic radiosurgery system that has a 6MV linear accelerator mounted on a computer controlled robotic arm and an orthogonal pair of diagnostic X-ray imaging devices. It can irradiate the target using 1200 points in the room [10–12], thereby, has the advantages to delivering higher doses to the target while avoiding doses to the normal structures.

In this study, we retrospectively analyzed the outcomes and prognostic factors affecting survival in 115 unresectable HCC treated with Cyberknife SBRT (Accuray Inc., sunnyvale, CA).

## Methods

### Patients

Between December 2008 and November 2012, 115 patients with unresectable HCC were treated with Cyberknife SBRT. Patients were included based on the following criteria (1) Pathological confirmation of HCC, (2) At least one radiological image showing the classic HCC enhancement with alpha fetoprotein (AFP) >200 ng/ml or at least 2 radiological findings (CT/MRI/Angiogram) showing the classic HCC, (3) the presentation of unresectable or medically inoperable HCC, and (4) ECOG performance status of ≤ 2. Patients with multiple extrahepatic metastases, previous radiotherapy for liver tumors, SGOT and SGPT levels of ≥ 2.5 times higher than the upper limit, Child-Pugh score of ≥ 7, intractable ascites, tumor closely attached to the esophagus, stomach, duodenum and bowel, and a liver volume of less than 700 cc were excluded from the study.

Mandatory elements included in the baseline examination are liver dynamic magnetic resonance imaging (MRI) and/or Triphase computed tomography (CT), complete blood study, liver function test, hepatitis B and C virus testing, alpha-fetoprotein (AFP), and chest images. Patients with HbsAg positive results or elevated hepatitis B virus DNA were given prophylactic anti-retroviral therapy from the start of SBRT to at least 6 months after the treatment for prevention of reactivation of HBV after radiotherapy [13–15].

The characteristics of the 115 patients and disease variables at the time of radiation treatment are summarized in Table 1. Median follow-up was 15 months (2-60 months). Their age ranges from 31-91 years, with a median age of 66 years and male predominance. Tumors were mostly located in the right lobe. The maximum tumor diameter ranged from 1.8- 18 cm.

**Table 1:** Clinical features and survival of study participants (N = 115)

Clinical features	N (%)	Survival		
		1 yr.(%)	2 yrs. (%)	*p*
Gender				
Male	88 (76.52)	63.67	38.98	
Female	27 (23.48)	62.96	57.89	0.523
Age (y.o)				
<60	37 (32.17)	56.76	29.63	
≧60	78 (74.26)	66.67	45.28	0.207
ECOG				
0	49 (42.61)	75.51	54.29	
1	53 (46.09)	58.49	36.36	
2	13 (11.3)	46.15	20	0.118
Child-Pugh score				
A	104 (90.43)	68.27	47.14	
B	11 (9.57)	27.27	12.5	<0.001
AJCC stage (7th)				
I	21 (18.26)	85.71	78.57	
II	18 (15.65)	72.22	60	
IIIA	26 (22.61)	69.23	40	
IIIB	27 (23.48)	37.03	11.11	
IIIC	3 (2.61)	100	0	
IV	20 (17.39)	55	37.5	0.001
BCLC				
A	12 (10.44)	83.33	75	
B	23 (20)	86.26	56.25	
C	80 (69.56)	55	31.48	0.080
Tumor type				
Solitary	38 (33.04)	81.58	62.5	
Multiple	69 (60)	56.52	31.25	
Diffuse	8 (6.96)	37.5	33.33	<0.001
Tumor site				
Right	91 (79.13)	64.83	42.86	
Left	14 (13.04)	53.33	38.46	
Bilateral	10 (8.7)	60	0	0.506
Max. tumor diameter (cm)				
≦4	40 (34.78)	75	55.17	
> 4-9	47 (40.87)	68.82	36.67	
≧10	28 (23.48)	48.15	26.32	0.187
Portal vein tumor thrombosis				
Yes	34 (29.56)	35.29	12	
No	81 (70.43)	75.31	53.7	<0.001
Hepatitis virus				
B	59 (51.3)	66.1	36.58	
C	42 (36.52)	59.52	46.43	
Non B non C	11 (9.57)	81.82	77.78	
B + C	3 (2.61)	66.67	0	0.576
AFP Level (ng/ml)				
≦20	41 (34.78)	82.93	62.5	
> 20-400	31 (27.84)	51.61	17.65	
≧400	43 (37.39)	53.49	34.48	0.013
Biochemical changes				
Albumin (g/dl) [N = 109]				
<3.5	26 (23.85)	46.15	31.81	
≥3.5	83 (76.15)	67.47	44.64	0.027
Alkaline Phosphatase (IU/L) [N = 104]				
≧129	37 (35.58)	59.45	42.57	
<129	67 (64.42)	64.18	36.95	0.893
Platelet (10^3/uL) [N = 109]				
<150	52 (47.71)	63.46	35	
≧150	57 (52.29)	68.42	48.65	0.291
BED				
≦72 Gy10	65 (56.52)	56.92	28.2	
73-88 Gy10	8 (6.96)	62.5	28.57	
≧89 Gy10	42 (36.52)	76.19	56.25	0.038
Previous treatment				
Yes	52 (45.22)	50	37.14	
No	63 9(54.78)	55.56	43.18	0.584

*Abbreviations*: *ECOG* Eastern Cooperative Oncology Group, *AJCC* American Joint Cancer Conference, *BCLC* Barcelona Clinic Liver Cancer Stage, *AFP* Alpha-Feto Protein, *BED* Biological Effective Dose

Patients were explained the advantages and disadvantages of cyberknife SBRT and made final treatment decision for themselves. Written informed consent was obtained from all patients before treatment, and the study was approved by the institutional review board of Chi Mei Medical Center.

#### SBRT

SBRT was performed using the Cyberknife, a robotic image-guided whole-body radiosurgery system with the synchrony respiratory tracking for targets that move with respiration. Synchrony accuracy is less than 1.5 mm for mobile targets, with a treatment accuracy of 0.3 mm [16]. 4 gold fiducial markers were implanted percutaneously around the perimeter of the target volume using a sono-guided procedure 5-7 days before the planning CT-scan. A Contrast simulation CT with slice thickness of 1-mm was performed covering the whole liver and bilateral kidneys. No Respiratory control and abdominal compression was used. The image data were then transferred to the Cyberknife system’s treatment planning workstation. Contouring was performed on the planning CT images with contrast. To better delineate tumor volumes, a set of MRI of liver was arranged in all patients, hepatic or delayed phases of MRI were fused with the planning CT scan for contouring, other phase images of MRI were used as a visual reference. The system automatically determined optimal beam directions and beam weights in order to maximize the dose delivered to the target and minimize that to the organs at risk. All patients were positioned on individually shaped vacuum pillows and wore vests to which the optical markers were attached. The displacement of the patient during treatment was tracked by either internal or external fiducial markers with sub-millimeter accuracy [10].

### Dose specification and plan evaluation

Prescribed doses, dose per fraction and number of fractions were individualized based on size and location of tumors, amount of normal liver and organs at risk. SBRT doses ranging from 26 to 40 Gy in 3-5 fractions were delivered to tumors > 4 cm and 39 Gy in 3 fractions to tumor ≤ 4 cm. The maximum diameter of tumors ranged from 1.8-18 cm. The SBRT doses was converted into normalized total dose at a fraction size of 2 Gy (NTD2Gy) using the linear quadratic equation (BED = total dose x (1 + dose per fraction/ α/β), α/β = 10 for early responding tissue, α/β = 3 for late responding tissue). NTD2 Gy ( α/β = 10) for SBRT ranges from 48.36 Gy to 89.70 Gy. The gross tumor volume (GTV) included diseases seen on contrast-enhanced CT or MRI scans. No CTV was further added. The GTV was directly expanded to 1-3 mm in all directions to create the planning target volume (PTV). Modification of PTV was done if it extended to the dose-limiting organs, except the normal liver. The prescription isodose line ranged from 59.9-96.9 % of maximum dose and median isodose line was 79.93 %. The radiation treatment was delivered with the real-time tracking system guided by fiducial markers, using the MultiPlan Treatment Planning System (version 2.10).

The dose constraints of the protocol for normal liver (total liver minus cumulative GTV) were specified that a minimum volume of 700 ml should receive a total dose less than 15 Gy [17]; 66.7 % of the volume delivered to the ipsilateral kidney should be less than 15 Gy; the maximum total dose to any point in the spinal cord should not exceed 18 Gy, 30 Gy to the stomach, bowel, duodenum and heart, and 27 Gy to the esophagus [18]. Efforts were made to minimize the dose to the normal tissues as much as possible.

### Follow-up, response, and toxicity assessment

After completion of treatment, the vital sign evaluation, physical examination, liver function test, and complete blood test were done to assess acute toxicity. They were followed every 1 to 2 weeks for the first month and every 3 months thereafter. Four-phase CT-scans or dynamic MRI of liver and AFP were obtained 1-2 months and, subsequently, every 3 to 4 months. Toxicity grading was according to the Common Toxicity Criteria Adverse Events, version 4.0. Acute toxicities were defined as adverse events within 3 months after SBRT, and late toxicities were those occurring after 3 months. Radiation-induced liver disease was defined as either classic or nonclassic RILD. Classic RILD was the presence of nonmalignant ascites and anicteric elevation of alkaline phosphatase level (twice the upper normal level) or baseline levels occurred between 2 weeks and 3 months after the completion of irradiation. Nonclassic RILD, typically occurring between 1 week and 3 months after therapy, involves elevation of transaminase to at least 5 times the upper limit of the normal or pretreatment levels within 4 months after completion of irradiation or decline in liver function in the absence of classic RILD [17, 19], which are the common endpoints among HCC patients with poor liver function (hepatitis B infection, Child-Pugh Classic B and C). The diagnosis of both RILD could be made only in the absence of evidence of tumor progression. Toxicity grading was based on the worst toxicity recorded.

Tumor response was assessed as described in the Response Evaluation and Criteria in Solid Tumors (RECIST) after completion of SBRT. Complete disappearance of the tumor was defined as complete response (CR), a decrease of more than 30 % in the longest diameter of target tumors as partial response (PR), a decrease of less than 30 % or no change as stable (SD), and progression of more than 20 % as progressive disease (PD). Local recurrence-free survival was divided into In-field and out-field intrahepatic recurrence-free survival, with the In-field being defined as no new lesion development or no increase in tumor size within the PTV and the Out-field as no new liver lesions outside the PTV. Distant metastasis was defined as recurrence outside the liver; disease progression was defined as the development of In-field or out-field intrahepatic recurrence and distant metastasis.

### Statistical analysis

The Overall survival rate (OSR) and Disease-Progression free survival (DPFS) were estimated from the commencement of SBRT to the last follow-up using the Kaplan-Meier method. The log-rank test was used to compare the survival curves over the period of follow-up time. Univariate hazard ratio (HR) with 95 % confidence intervals were estimated by Cox proportional hazards regression. Significant factors in univariate Cox analysis were applied to the multivariate Cox proportional hazards regression analysis based on the Collett's model selection approach.[20] Analysis of data was performed using SPSS (SPSS Inc., Chicago, IL, USA) version 17 software. The statistical significant level was set at p value <0.05.

## Results

### Tumor response and local control

The tumor response was evaluated by the change in maximal diameter of the tumor on CT-scan or MRI 4-6 weeks after treatment completion, followed by every 2 to 3 months subsequently. As summarized in Table 2, an objective response was observed in 102 of 115 patients (88.7 %). 56 (48.7 %) and 46 (40 %) patients achieved complete and partial response, respectively; stable disease was observed in 10 (8.7 %) patients; tumor progression was seen in 3 (2.61 %) patients. Patients achieving complete response had a significantly favorable survival. 1- and 2-yr OSR of patients with complete response was 87.5 % and 68.6 %, with median survival rate of 21 months (13.5-31 months). The OSR of patients with stable disease was also far better than those achieving partial response. The 1- and 2-yr OSR of patients with stable disease was 70 % and 50 %, respectively, with median survival being 20.5 months (10-27 months); 1- and 2-yrs OSR of patients with partial response was only 39.1 % and 12.5 %, with median survival rate of 9 months (5-16 months). Patients with tumor progression had the worst outcome (none survived more than 1 year) with median survival of only 4 months (1-7 months).

**Table 2:** Tumor Response, RECIST (N = 115)

Clinical feature	N (%)	Survival Rate		*p*
		1-yr (%)	2-yrs (%)	
Complete Response	56 (48.7)	87.5	68.57	
Partial Response	46 (40)	39.13	12.5	
Stable	10 (8.7)	70	50	
Tumor progression	3 (2.61)	0	0	0.001

**Fig. 1:**
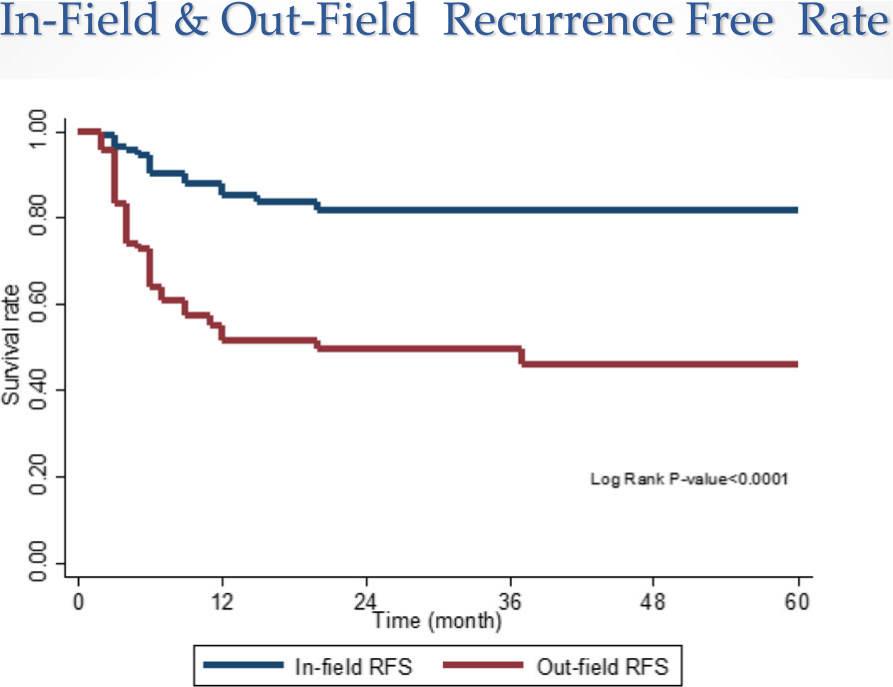
In-field recurrence free rate at 1-yr and 2-yrs were 85.3 % (76.2-91.1) and 81.6 % (71.2-88.6), respectively. Out-field recurrence free rate at 1-yr and 2-yrs were 51.5 % (41.2-60.8) and 49.5 % (38.9-59.2), respectively

Local control was divided into In-field and out-field recurrence free (IFRF & OFRF) groups. The 1- and 2-year of IFRF rate were 85.3 % (76.3-91.1) and 81.6 % (71.2–88.6), while those of OFRF were 51.5 % (41.2-60.8) and 49.5 % (38.9 -59.2) at 1-and 2-year, respectively.(*p* = < 0.0001, Fig. 1). Tumor size (≤4 cm vs. ≥4-9 cm) was the only significant predictor for the in-field recurrence free (*p* = 0.041) rate. In contrast, biological effective dose (≤72 Gy vs. ≥ 89 Gy, *p* = 0.023) and tumor location (R vs. bilateral lobe [R/L], *p* = 0.041) were statistically significant factors associated with out-field recurrence free rate (Table 3).

**Table 3:** Prognostic factors: Univariate and Multivariate analysis

Parameter	Overall survival		In-field recurrence free		Out-field recurrence free	
	UA	MA	UA	MA	UA	MA
		HR (95%CI) P		HR (95%CI) P		HR(95%CI) P
M vs F	0.531	NS	0.377	NS	0.150	NS
Age (y.o)						
<60 vs ≧60	0.217	NS	0.552	NS	0.195	NS
ECOG						
0 vs 1	0.052	NS	0.439	NS	0.910	NS
0 vs 2	0.234	NS			0.620	NS
AJCC Stage (7th )						
1 vs 2	0.584	NS	0.833	0.974 (0.054-17.559) *0.986*	0.380	0.955(0.281-3.251) *0.941*
1 vs 3a	0.058	NS	0.240	1.189 (0.108-13.124) *0.888*	0.010	1.684(0.540-5.253) *0.369*
1 vs 3b	0.001	NS	0.021	5.179 (0.320-83.713) *0.247*	0.009	1.549(0.495-4.847) *0.452*
1 vs 4	0.063	NS	0.396	0.730 (0.053-10.142) *0.815*	0.506	0.841(0.228-3.099) *0.795*
BCLC						
A vs B	0.502	NS			0.066	NS
B vs C	0.077	NS			0.092	NS
Portal Vein Tumor Thrombosis						
N vs Y	<0.001	1.774 (1.037-3.036) *0.036*	0.035	0.976 (0.167-5694) *0.978*	0.054	NS
Child Pugh score						
A vs B	0.001	3.183 (1.397-7.255) *0.006*			0.221	NS
Hepatitis virus						
B vs C	0.766	NS	0.663	NS		
B vs Non B/C	0.766	NS	0.57	NS		
B vs B + C	0.225	NS				
Albumin (g/dl)						
≥3.5 vs <3.5	0.033	NS	0.099	NS		
Alkaline Phospatase (IU/L)						
≥129 vs < 129	0.895	NS	0.999	NS	0.405	NS
Platelet (10^3/uL)						
≥150 vs <150	0.302	NS	0.847	NS	0.441	NS
BED						
≦72 vs 73-88	0.980	NS			0.808	0.944 (0.316-2.823) *0.918*
≦72 vs ≥89	0.016	NS	0.052	NS	0.003	0.423 (0.201-0.890) *0.023*
Tumor Size (cm)						
≦4 vs >4-9	0.428	2.665 (1.437-4.946) *0.002*	0.027	9.210 (1.095-77.473) *0.041*	0.872	NS
≦4 vs ≧10	0.085	4.928 (1.794-13.546) *0.002*	0.034	6.371 (0.630-64.451) *0.117*	0.141	NS
Tumor Type						
Solitary vs Multicentric	0.003	NS	0.042	4.173 (0.790-22.044) *0.093*	0.003	2.084 (0.880-4.933) *0.095*
Solitary vs Diffuse	<0.001	NS	0.243	4.994 (0.338-73.688) *0.242*	0.674	0.973 (0.186-5.094) *0.974*
Location						
R vs L	0.903	NS	0.816	NS	0.722	1.231 (0.528-2.872) *0.631*
R vs R/L	0.257	NS	0.074	NS	0.006	2.545 (1.038-6.238) *0.041*
AFP (ng/ml)						
≦20 vs 20-400	0.005	NS	0.070	NS	0.360	NS
≤20vs ≥400	0.066	NS	0.117	NS	0.985	NS
Previous treatment						
No vs Yes	0.592	NS	0.318	NS	0.821	NS

Abbreviations as in Table 1

a. Identify the predictors significant at *p*-value < 0.2. b. The effects met the 0.1 level for forward and backward method entry into the multivariate survival model based on Collett’s model selection approach. c. *p*-value of <0.05 was significant

### Overall survival and prognostic factors

**Fig. 2:**
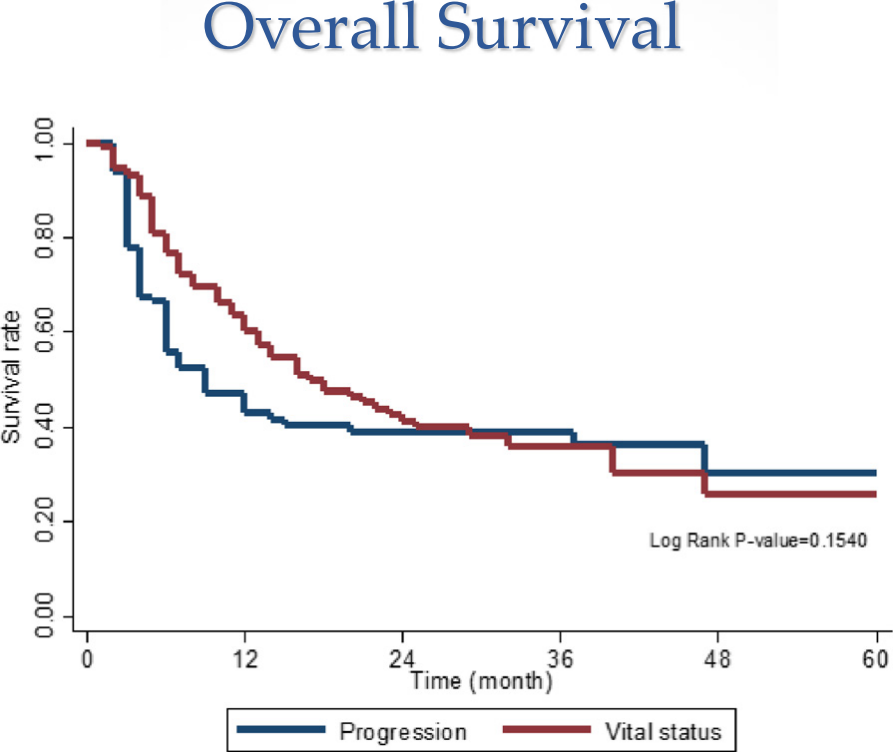
Overall survival curve of 115 patients treated with Cyberknife SBRT. 1-yr and 2-yrs OSR were 63.5 % (54-71.5 %) and 41.3 % (31.6-50.6 %), median survival times was 15 months (4-25 mos.), While 1-yr and 2-yrs Progression-free survival rate were 42.8 % (33.0-52.2 %) and 38.8 % (29.0-48.4 %), median progressive-free survival times was 6 months, respectively

The median follow-up time was 15 months (range, 2-60 months). The overall survival rate (OSR) for 1 and 2 years was 63.5 % (54-71.5 %) and 41.3 % (31.6-50.6 %), with median survival time of 15 months (4-25 months); the 1- and 2-year progression-free survival rates were 42.8 % (33.0-52.2 %) and 38.8 % (29.0-48.4 %), with median progressive-free survival time of 6 months, respectively (Fig. 2). Table 1 shows the clinical features and survival of the participants.

The analysis of the prognostic factors was based on survival calculated from the start of SBRT. Multivariate analysis demonstrated that portal vein tumor thrombosis (yes vs. no), Child-Pugh score (A vs. B), tumor size (≤4 cm vs. > 4-9 cm and ≥ 10 cm), and tumor response after SBRT (CR vs. PR and DP) were the independently significant predictors of OS (Table 3).

### Toxicities

Acute toxicities are listed in Table 4. In general, SBRT is tolerable. Grade 1-2 Fatigue was the most common sequelae and was developed in 59.13 % of the patients. The other common toxicity was alteration in liver function test, especially SGOT (56.52 %) and SGPT (49.56 %). These effects were usually grade 1 or 2 and transient, and most patients eventually recovered 2-4 weeks later. Thrombocytopenia was found in 65 patients (61.74 %), 46 patients (40 %) had decreased hemoglobin levels, and 15 (13.04 %) had leukopenia. These toxicities were also transient and tend to recover to their previous levels 4 weeks later. Other sequelae include grade 1-2 chest wall pain in the lower right side found in 20 patients (17.39 %) and grade 1-2 dermatitis in 3 patients (2.61 %). These were frequently found in patients with HCC closely adhered to adjacent ribs and skin. In order not to compromise the PTV coverage, rib and skin constraints were not considered. Fortunately, no severe complication (more than grade 2) was observed. Aside from ≤ grade 2 nausea and vomiting (22.61 %), there was no other acute gastrointestinal toxicity such as gastroduodenal ulcer and gastroenteritis colitis.

**Table 4:** Toxicity, CTCAE v 40

Toxicity	Grade 1		Grade 2		Grade 3		Grade 4		Grade 5		Total
	No.	%	No.	%	No.	%	No.	%	No.	%	%
Fatigue	64	55.65	2	1.74	1	0.87	0	0	0	0	59.13
Pain (RUQ/ chestwall)	18	15.62	2	1.74	0	0	0	0	0	0	17.39
Biochemical											
SGOT	49	42.61	8	6.96	7	6.09	0	0	1	0.87	56.52
SGPT	42	36.52	8	6.96	6	5.22	0	0	1	0.87	49.56
Alk.P	36	31.3	3	2.61	4	3.48	0	0	0	0	37.3
Bilirubin	12	10.43	3	2.61	1	0.87	0	0	1	0.87	14.78
Albumin	38	33.04	6	5.22	0	0	0	0	0	0	38.26
Hematologic											
Anemia	44	38.26	2	1.74	0	0	0	0	0	0	40
leukopenia	9	7.83	6	5.22	0	0	0	0	0	0	13.04
Thrombo-cytopenia	45	39.13	20	17.39	6	5.22	0	0	0	0	61.74
Nausea/ vomiting	24	20.87	2	1.74	0	0	0	0	0	0	22.61
Dermatitis	1	0.87	2	1.74	0	0	0	0	0	0	2.61

Eight patients experienced ≥ grade 3 liver function alteration within 3 months of SBRT. Five of them were caused by disease progression and three were nonclassic RILD. No classic RILD was observed. Among the three patients with nonclassic RILD, two had underlying HCV cirrhosis and one had HBV cirrhosis; 2 were BCLC stage C and 1 was BCLC stage B; all three belonged to Child-Pugh A. Two of them eventually recovered to their previous levels 1- 3 months after SBRT. However a 54-year-old male patient with cT3bN0M0 HCC, HBV liver cirrhosis, Child-Pugh A , went into liver failure and died 2 months later. Cyberknife SBRT with 40 Gy in 5 fractions was given for treatment after failure of TACE. HBV DNA level before treatment was 1,560,000 copies/ml. Antiviral drugs were prescribed to the patient but he did not comply with medication treatment. Reactivation of HBV DNA level to 1,610,000,000 copies/ml was noted 3 weeks after SBRT, with rapid elevation of transaminase level to 1885 IU/L , other liver function datas simultaneously deteriorated. However, image studies from abdomen sonography and MRI of liver shows partial regression of previously treated liver tumor and portal vein tumor thrombosis, with no evidence of new lesion. Supportive treatment was given but the patient eventually died from liver failure 4 weeks later.

## Discussion

The results of this study support the fact that Cyberknife SBRT could be a treatment option for patient ineligible for local ablation therapies or surgical resection. A large proportion of our study population had locally advanced disease or recurrence after standard treatment and difficult-to-reach tumors. In comparison with other series, in which SBRT is rarely used on tumor larger than 7 cm, the median size of the largest tumor in the current study was 18 cm. Huge tumor (≥10 cm) comprise 23.48 % of the cases, while portal vein tumor thrombosis was present in 29.56 % of patients. In a large prospective study of 102 patients with locally advanced HCC treated with a six-fraction SBRT regimen, maximum tumor size was less than 7.2 cm. Bujold et al. reported a 1-year local control rate of 87 % [3]. Mendez-Romero et al first described the results of prescribing 25 to 37.5 Gy in three to five fractions to 25 patients with inoperable HCC and liver metastases in another prospective study. Median lesion size was 3.2 cm. (range 0.5-7.2 cm). Local control rate at 1 and 2 years were 94 % and 82 % [21]. In our study, despite the large number of patients with large tumor burden (40.87 % patient was >4-9 cm, 23.48 % patient was ≥ 10 cm) , the 1-year and 2-year in-field recurrence free rate of 85.3 % and 81 %, were encouraging. Tumor size was the most significant factor affecting local control. Most HCC is a multicentric disease and the patients tended to have a higher risk of local recurrence after treatment [22]. Despite high in-field recurrence free after SBRT in our study, similar to other treatment modalities, intrahepatic out-field recurrence remains the major problem. Thus, combination of SBRT and systemic therapies is reasonable. A recent retrospective analysis of 23 patients with advanced HCC treated with concurrent hypofractionated radiation therapy (52.5 Gy in 15 fractions) with sunitinib (Sutent;Pfizer, New York, NY) demonstrated prolongation of time to progression from 4 months to 10 months [23]. However, an early phase 1 study combining SBRT with concurrent sorafenib by the University of Toronto suggested that higher dose of sorafenib (400 mg daily), when combined with radiation, could delivered higher values of effective liver volumes (Veff 30 %-60 %) irradiated, yielding a significant (grade 3+) toxicity [24]. An ongoing RTOG 1112 phase 3 study of sorafenib versus SBRT followed by sorafenib in HCC is being conducted. In this study, sorafenib will be delivered after completion of radiation, rather than concurrently with radiation, to reduce the risk of treatment toxicity.

In our study, 1- and 2-yr OSR was 63.5 % (54-71.5 %) and 41.3 % (31.6-50.6 %), respectively. Median survival was 15 months and median progressive-free survival was 6 months. Objective response (CR + PR) of 88.7 % compared favorably with best supportive care, even better than sorafenib and sunitinib, which are the only other options for this patient population. In a randomized phase 3 trial of sunitinib vs. sorafenib for advanced HCC by Cheng et al., the median survival for sunitinib was 7.9 months while sorafenib was 10.2 months; the median progression-free survival was 3.6 vs. 3.0 months [25]. One other study by Llovet et al. reported a 1-yr OSR of 44 % and median survival of 10.7 months for sorafenib patients [6]. A large randomized trial of Sorafenib from Asia-Pacific region showed a median overall survival of only 6-5 months and median to progression of 2.8 months [7].

Consistent with other studies, portal vein tumor thrombosis remains the established prognostic factors in the study series. The median survival was 8 months vs. 17.5 months for HCC with and without PVTT; 1- and 2-year overall survival was 35.3 % and 12.50 % vs. 75.31 % and 53.7 %. A retrospective study by Xi et al. reported a median survival of 13 months and a 1-year overall survival of 50.3 % for 41 HCC patients with PVTT and/or with inferior vena cava thrombus treated to a median dose of 36 Gy in 6 fractions targeting the tumor thrombus [26]. Another prospective study from University of Toronto reported a median survival of 10.6 months versus 21.5 months for HCC with and without PVTT, respectively, with a total radiation dose ranging from 24-54 Gy in 6 fractionations schema [3]. Compared with other studies series using SBRT for HCC with PVTT, a larger proportion of our patients with PVTT (29.56 %) had a large tumor burden (>4-9 cm in 40.87 % of patients, ≥ 10 cm in 23.48 % of patients) and 66.9 % of tumor types were multicentric and diffuse in our present cohort, which partly explain why OS was lower than expected in our study. Child-Pugh score, tumor size and tumor response are other major independent risk factors for overall survival. Tumor size is the only independent predictive factor for In-field recurrence free rate. Patients with tumors ≤ 4 cm had significantly better outcomes than those with > 4 cm and ≥ 10 cm tumors. Tumors located mainly in the right lobe and a higher biologically effective dose were the major predictive factors for out-field recurrence free rate. In our study series, BED 89 Gy (39 Gy in 3 fractions) was given to small (≤4 cm.), solitary and peripheral tumors, expectedly to have a better local control and less chance of developing a multicentric type of HCC. In an earlier study from RTOG report, a high-dose group showed better results [27]. Park et al, show a dose-response relationship in the local control rate of primary HCC [28]. Another study by Park et al, reported that BED_10_ > 50 Gy had a significant better response rate (complete or partial response) of 72.8 % compared with 46.7 % with BED_10_ ≤ 50Gy (p = 0.0299) [29]. While possible hypothesis for higher chance of out-field recurrence free rate from higher BED10, is the enhanced antitumor immunity after SBRT of tumor at 1 site contributes to rejection of metastatic lesion at distant sites, so called abscopal effect reported by Postow et al [30]. However, the clinical evidence was found only in two Melanoma patients. Another preclinical study on mice by Lee et al [31] has reported a similar enhancement of antitumor immunity after high dose irradiation of local tumor. These results are clearly exciting but much more information is needed to recommend the most optimal radiation therapy treatments.

The use of Cyberknife SBRT to treat primary HCC is an important aspect of our study. In comparison with conventional fractionated RT, Cyberknife SBRT demonstrates its benefits for achieving highly conformal dose distributions with respiratory synchronization while sparing the adjacent normal liver [32]. This allows higher biologically effective dose to be delivered without increased incidence of liver toxicities, and better local control may be achieved. In terms of toxicity, the present study shows that Cyberknife SBRT is feasible and safe in primary HCC patients, which is consistent with the findings of previous SBRT study [6, 21]. Of the 3 documented cases of RILD in our study, 2 eventually recovered and one died as a result of radiation-induced liver failure 2 months after treatment. And the cause of RILD was the reactivation of hepatitis B virus. Kim et al demonstrated that 3D-CRT can induce HBV reactivation in patients with HBV-related HCC. They defined elevated transaminase level, 2.5 fold the upper limit of normal accompanying an increase of > 2 log_10_ copies /mL in HBV DNA as a criteria for HBV exacerbation in patient undergoing 3D-CRT for HBV-related HCC [13] . Cheng et al, found that HCC patients with underlying hepatitis B virus carriers or Child-Pugh B cirrhosis showed signifcantly greater susceptibility to radiation-induced liver disease after three-dimensional conformal radiotherapy. Asian patients with HCC usually have viral hepatitis, therefore, dose distribution in liver is crucial to the preservation of liver function [15]. Huang et al. and Janoray et al. reported the RILD incidence rate of 5.5 % and 0 or 9 %, respectively, using Cyberknife SBRT [33, 34]. Earlier reports by Takeda et al. and Tse et al. also reported no serious SBRT-related toxicities [8, 35]. In contrast, there were much more treatment-related complications with other local therapies. In cases of TACE, rate of acute liver failure is approximately 7.5 % and treatment related mortality rate is 2.4 % after TACE [36]. For RFA, major complications developed in 2.2 %-12.6 % of patients, and the mortality rate is 0-0.8 % [37]. RILD incidence was low and most cases were reversible after Cyberknife SBRT for HCC. However, further prospective studies might be required to determine the predictive factors of radiation-induced liver disease and optimal dosing regimen.

The major limitation of our study is that it was a retrospective single-institution study with small and hetergenous sample size. However, this is the largest study to date that focused on the use of Cyberknife SBRT for inoperable HCC.

## Conclusions

In the present study, excellent in-field control was obtained for Cyberknfie SBRT in HCC. In-field recurrence free rate at 2-years was 81.6 %, tumor response rate (CR + PR) was 88 %, and 1- and 2-year overall survival rates were 63.5 % and 41.3 %, which was encouraging. The acute toxicity was relatively mild and tolerable. In a population of patients for whom curative local treatment is not applicable, SBRT can lead to sustained local control and a higher survival rate than historical controls, with a low risk of morbidity. Out-field recurrence is still the major cause of failure, providing a rationale for combining SBRT and regional or systemic therapies. Further study is required to define the optimal radiation dose and fractionation for future SBRT treatment.

## Abbreviations

SBRT, Stereotactic body radiation therapy; HCC, Hepatocellular carcinoma; TACE, Transarterial Chemoembolization; RT, Radiation therapy; MRI, Magnetic resonance imaging; CT, Computed tomography; BED, Biological effective dose; GTV, Gross Tumor Volume; PTV, Planning Target volume; RILD, Radiation-induced liver disease; CR, Complete response; PR, Partial response; SD, Stable; PD, Progressive disease; OSR, Overall survival rate; DPFS, Disease-Progression free survival; IFRF, In-field recurrence free; OFRF, Out-field recurrence free; BCLC, Barcelona clinic liver cancer stage; HCV, Hepatic C virus; HBV, Hepatic B virus; RFA, Radiofrequency tumor ablation; 3D-CRT, three dimensional conformal radiation therapy
